# Pixelated Metasurface for Dual-Band and Multi-Polarization Electromagnetic Energy Harvesting

**DOI:** 10.1038/s41598-018-31661-6

**Published:** 2018-09-05

**Authors:** Bagher Ghaderi, Vahid Nayyeri, Mohammad Soleimani, Omar M. Ramahi

**Affiliations:** 1 Antenna and Microwave Research Laboratory and School of Electrical Engineering, Iran University of Science and Technology, Tehran, 1684613114 Iran; 2 Antenna and Microwave Research Laboratory and School of New Technologies, Iran University of Science and Technology, Tehran, 1684613114 Iran; 3 Department of Electrical and Computer Engineering, University of Waterloo, Waterloo, Ontario N2L3G1 Canada

## Abstract

A dual-band and polarization-independent electromagnetic energy harvester composed of an array of pixelated unit cells is proposed. The pixelated unit cell is basically a dual-band resonator loaded with two resistors which model the input impedance of a power combining circuit in a complete harvesting system. To design the unit cell, a topology optimization approach based on pixelization of the surface of the unit cell and application of a binary optimization algorithm is used. The optimization goal is set to maximize harvesting efficiency at 2.45 GHz and 6 GHz. In our design, full symmetry of the unit cell is considered to achieve insensitivity to the polarization of the incident wave. Once, the unit cell is designed, as a proof of the concept, a metasurface harvester composed of 9 × 9 pixelated cells is designed. The full-wave electromagnetic simulation results demonstrate that the proposed metasurface absorbs the incident electromagnetic wave energy with nearly unity efficiency at both frequencies of interest and irrespective the polarization of the incident field while simultaneously delivering the absorbed power to the loads. To validate the simulations, the metasurface harvester is fabricated and tested in an anechoic chamber. A strong agreement between the simulation results and measurements is observed.

## Introduction

Recently, energy harvesting (and its applications in wireless power transfer) has attracted strong interest due to its potential to improve the mobility and reliability of low power wireless devices^1^. Within the frequency spectrum, electromagnetic (EM) energy harvesting in radio frequency (RF) and microwave regime has interesting features including low cost, feasibility of long-range power transfer and compact size^2^. Every RF and microwave harvesting system includes a rectifying antenna (rectanna) which captures the radiated EM wave from the ambient and then converts the captured power to DC^3,4^. Recently arrays of electrically small resonators such as split-ring resonator (SRR)^5^ and complementary split-ring resonator (CSRR)^6,7^, namely metasurfaces (or metamaterials) have been shown to be promising alternatives to conventional antennas with the key advantage of higher efficiency. Similar to metasurface absorber^8–18^, in a metasurface harvester, the small resonators, in their resonance frequency, effectively couple to the incident EM wave and capture the EM power from the ambient. While in absorbing functionality, the captured EM power dissipates within the structure as either ohmic or dielectric loss^8–12^, in harvesting application, the captured power by each resonator is chanelled to a rectifying circuit through a network which combines the power captured by one or multiple resonators^19–21^. The input impedance of each branch of a power combiner can be modeled by a resistive load (a resistor with one grounded port); therefore, in metasurface harvester designs, each unit cell is commonly loaded with one (or more) grounded resistors^6,7,19–23^. On the other hand, in some recent metamaterial absorber designs, the absorbed power dissipates in lumped resistors which are placed between two sections of a resonator^13–18^. These structures cannot be applied as harvester because the resistors are not grounded, therefore, replacing them with a power combining network is challenging.

Several works have improved the performance of metasurface EM energy harvesters which include increasing the harvesting bandwidth by introducing an array of bow-tie CSRRs^22^ and enhancing the harvesting efficiency up to near unity using an ensemble of electric-inductive-capacitive (ELC) resonators^23^. Recently, multi-band and multi-polarization metasurface harvesters have been proposed^21,24–26^. It should be noted that, a design with multi-polarization feature is capable to harvest the EM power irrespective to the polarization of the incident wave, hence improving the functionality of energy harvesting. A multi-band design, on the other hand, enables harvesting from several radiation sources with different frequencies.

Recently, a triple-band polarization-insensitive metasurface based on SRRs was introduced where each cell consists of four identical SRRs with each loaded with a resistor arranged in a central symmetry^24^. A harvesting efficiency of 30%, 90% and 74% at 1.75 GHz, 3.8 GHz and 5.4 GHz, respectively for different polarization angles was achieved. Since the captured power by each cell is divided between four loads and considering that in a practical harvesting system the absorbed power must be combined, these designs call for an increase in the number of loads, which results in a more complicated and more lossy combining network. More recently, a metasurface composed of an array of sub-wavelength butterfly-shaped closed ring resonators was proposed for triple-band polarization-insensitive EM energy harvesting^25^. A harvesting efficiency of 90%, 83% and 81% at the frequency bands of 0.9 GHz, 2.6 GHz and 5.7 GHz, respectively, was reported. However, to achieve such a high efficiency, the load resistance was required to be very high (around 3 kΩ) which differs considerably from the input impedances of microwave power combining networks, hence restricting the application in a real-world harvesting system. Most recently, in two parallel works, multi-polarization EM energy harvesting in the frequency band of 2.4 GHz was achieved using arrays of ELC resonators^21,26^. In these designs, each unit cell is connected to one and two terminals with input impedance of 150 Ω and 200 Ω^21,26^. As shown theoretically and experimentally in^21^, because of the proper values of the input impedance of the terminals, they can be be simply connected to a power combining network.

In this work, we applied a complete-cycle procedure for design of dual-band polarization-insensitive metasurface harvester. Our design method is based on pixelization of the surface of a unit cell and then application of a binary optimization algorithm to maximize the harvesting efficiency at 2.45 GHz and 6 GHz. Such a versatile topology optimization approach (with different design goals) has been used for designing frequency selective surfaces^27^, high impedance surfaces^28^ and radar cross section reducer metasurfaces^29^. The designed metasurface harvester provides a harvesting efficiency of more than 90% in both frequency bands of interest for any polarization angle of the incident wave which is validated by performing laboratory measurements. We emphasis that, in this paper, similar to most earlier works on design of metasurface harvesters^5–7,22–26^, we focus on the RF (or AC) harvesting efficiency without considering the loss imposed by the combining and rectifying circuits. However, it has been theoretically and experimentally shown that implementation of a metasurface harvester in a complete harvesting circuit including a combining network and a rectifying circuit is feasible^19–21^. The challenges and considerations for design and fabrication of combining network for metasurface harvesters with a great number of cells are addressed in these works^19–21^. The advantages of the present work over the earlier multi-band multi-polarization designs^24,25^ are: (1) Each cell of our metasurface array has only two terminals whereas in^24^ each cell is connected to four terminals requiring a more complex power combining network. (2) To achieve a maximum harvesting efficiency, the impedance of the terminals are required to be 240 Ω, hence, the terminals can be simply connected to a power combining network. In^25^ the impedance of the terminals is required to be around 3 kΩ.

## Preliminary Design: A Simple Unit Cell for Dual-Band EM Energy Harvesting

**Figure 1:**
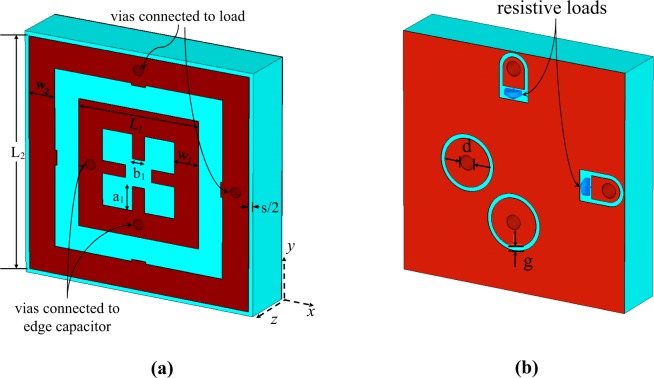
Proposed dual band unit cell structure, (**a**) top view and (**b**) bottom view ((**b**) is a mirror image of the actual view).

Figure 1 shows a new proposed metasurface unit cell for dual-band multi-polarization energy harvesting. As shown in Fig. 1(a), the unit cell consists of a ring resonator and a symmetric ELC resonator^21,26^, hosted on a Rogers RT/duroid 6006 substrate with a dielectric constant of 6.15, a loss tangent of 0.0027 and a thickness of 2.5 mm. It should be noted that a very low-loss substrate was used to minimize the dielectric loss which degrades the harvesting efficiency. The ring resonator is loaded with two resistive loads, which model input impedance of a power combining network, through two vias, while, the symmetric ELC resonator is loaded with two edge (gap) capacitance (see Fig. 1(b)). Using the full-wave frequency domain solver CST Microwave Studio^30^, the cell was truncated by a period boundary condition in the *x* and *y* directions and was excited by Floquet ports in a way that a plane wave normally illuminated the structure (incident in the -z direction). The simulation results showed that a periodic array of the unit cell exhibits a dual-band harvesting behavior, where the first operating frequency is adjusted by the length of the ring resonator (*L*_2_ in Fig. 1(a)), while the second operating frequency band is well controlled by the length of ELC resonator (*L*_1_ in Fig. 1(a)) and the gap capacitance between the disks and the ground plane (see Fig. 1(b)) which is determined by the gap size (*g*). Optimizing the design parameters shown in Fig. 1 to achieve a maximum harvesting efficiency at the frequencies of 2.45 GHz and 6 GHz resulted in *L*_1_ = 6 mm, *w*_1_ = 1.2 mm, *a*_1_ = 1.15 mm, *b*_1_ = 0.6 mm, *L*_2_ = 11 mm, *w*_2_ = 1.3 mm, *s* = 0.4 mm, *d* = 0.7 mm, *g* = 0.4 mm and the load resistance of *R* = 240 Ω.

**Figure 2:**
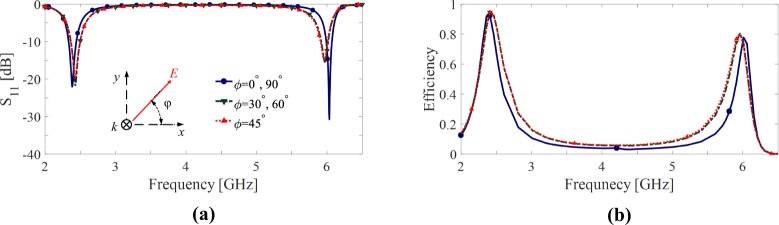
Reflection coefficient (**a**) and harvesting efficiency (**b**) of the proposed dual band unit cell shown in Fig. 1 for different polarization angles (**b**).

Figure 2(a), shows the magnitude of the reflection coefficient (*S*_11_) at the interface between the slab (i.e., an infinite periodic array of the unit cells) and the free space for different polarization angles (*ϕ*) of the incident wave as shown in the inset of Fig. 2(a), the polarization angle is defined as the angle between the electric field and the *x* axis. The figure shows that the slab is well matched to the free space (i.e. there is no considerable reflection at the interface) in both operating frequencies. On the other hand, since the structure is backed by a metal plate (see Fig. 1(b)) the transmission through the structure is almost zero as there is a negligible transmission due to the defects on the ground plane. Consequently, the absorption of the structure is defined as *A*(*ω*) ≈ 1 − |*S*_11_|^2^; therefore at the frequencies of 2.45 GHz and 6 GHz where the structure has a very low reflection, most energy of incident wave is absorbed by the structure. However, for the harvesting functionality, not only a very high absorption of the incident wave is required but also the absorbed energy is required to be channelized to the loads which model input impedance of a combining network. In other words, the harvesting efficiency is commonly evaluated as the ratio of the power delivered to the loads (*P*_*del*_) to the incident power (*P*_*inc*_),

1 $$\eta =\frac{{P}_{del}}{{P}_{inc}}.$$

Figure 2(b) shows the harvesting efficiency of the proposed unit cell for different polarization angles. Although, a relatively good harvesting efficiency in both operating frequency bands is achieved, efficiency in the second band is below 80%. Furthermore, as shown in Fig. 2(a), by changing the polarization angle of the incident wave, the resonance frequencies (specially the second one) of the cell slightly shift which causes a frequency shift in the peak of the harvesting efficiency of the cell in Fig. 2(b).

## A Pixelated Unit-Cell with Improved Performance

**Figure 3:**
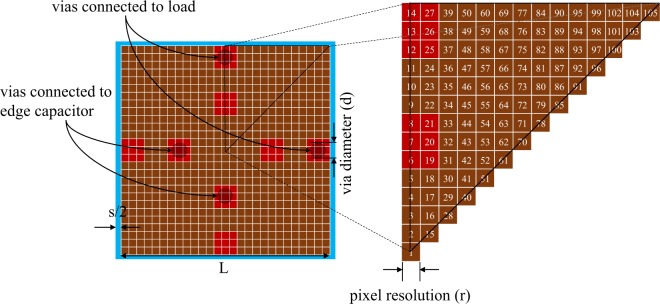
Pixelization of the unit cell.

To achieve a better harvesting performance in the frequency bands of interest (2.45 GHz and 6 GHz), a topology optimization routine is employed to obtain the best possible shape of the unit cell. To this end, as shown in Fig. 3, the size of the cell is set to (*L* + *s*) × (*L* + *s*) = 11.4 × 11.4 mm^2^ where *L* = *L*_2_ (in Fig. 1(a))  = 11 mm and *s* = 0.4 mm were obtained in the preliminary design of the cell. Then, as shown in Fig. 3, an area of *L* × *L* inside the cell is pixelated into 27 × 27 pixels with a resolution of 0.41 mm. Next, a binary value of 1 or 0 is assigned to each pixel (i.e. each pixel is represented as a bit) where having the value of 1 or 0 indicates the presence or absence of copper on the area of the pixel (on the top of dielectric substrate). Subsequently, by applying a binary optimization algorithm to the string of the bits, one can optimize the shape of the unit cell to achieve the desired goals. To make the design insensitive to the polarization of the incident wave, a four-fold symmetry (with respect to the lines that bisect the structure vertically, horizontally and diagonally) is enforced in the design of the unit cells. Therefore, only the pixels located in one-eighth of the pixelated area are involved in the optimization procedure (see Fig. 3). Furthermore, the bits representing the pixels which overlap the vias (see the red pixels in Fig. 3) are set “1”, i.e. open-ended vias are avoided.

Since, not only the shape of the cell but also the load resistance (*R*) and gap size (*g*) (see Fig. 2(b)) strongly influence the harvesting performance, simultaneous optimization of the shape of the cell and these values (i.e. *R* and *g*) is more efficient. However, optimization of real-valued *R* and *g* cannot be integrated with the binary optimization of the cell shape. Therefore, discrete values of *R* (100 Ω, 120 Ω, 150 Ω, 180 Ω, 200 Ω, 240 Ω, 270 Ω and 330 Ω) and *g* (0.1 mm, 0.25 mm, 0.4 mm and 0.55 mm) are considered and coded with 3 and 2 binary bits, respectively. Now, applying a binary optimization algorithm can optimize the shape of the cell and the values of load resistance and gap size simultaneously.

To accelerate the convergence of the optimization algorithm, the initial values of the bits were set such that the initial shape of the cell and initial values of load resistance (*R*) and gap size ( *g*) were those obtained from the preliminary design, i.e. Figure 1(a), *R* = 240 Ω and *g* = 0.4 mm.

**Figure 4:**
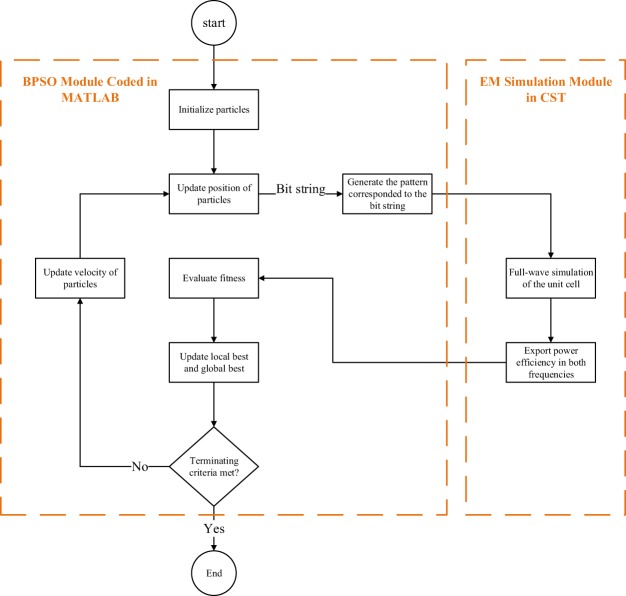
Flowchart of the design procedure.

Figure 4 shows the design flowchart of the pixelated cell. The binary particle swarm optimization (BPSO) algorithm^31,32^ which is a binary version of the PSO^33,34^, a well-known and robust global optimization method is implemented in a MATLAB code. In the PSO algorithm, a population (called a swarm) of candidate solutions (called particles) moves around the search space (in other words, each particle’s position is a candidate solution of the problem). In an optimization problem with *N* optimization parameters, the search space is N-dimensional and the position and velocity of mth particle are represented by two N-dimensional vectors **X**_*m*_ = [*x*_*m*,1_, *x*_*m*,2_, ..., *x*_*m*,*N*_] and **V**_*m*_ = [*v*_*m*,1_, *v*_*m*,2_, ..., *v*_*m*,*N*_], respectively. The particles adjust their velocity according to their own experiences and the best one explored by the swarm in the search space. At the tth iteration of the optimization algorithm, the velocity of the mth particle is determined as

2 $${{\bf{V}}}_{m}^{t}=w{{\bf{V}}}_{m}^{t-1}+{c}_{1}{e}_{1}({{\bf{P}}}_{m}^{t-1}-{{\bf{X}}}_{m}^{t-1})+{c}_{2}{e}_{2}({{\bf{G}}}^{t-1}-{{\bf{X}}}_{m}^{t-1}),$$

where **P**_*m*_ = [*p*_*m*,1_, *p*_*m*,2_, ..., *p*_*m*,*N*_] and **G** = [*g*_1_, *g*_2_, ..., *g*_*N*_] are the best experiences for the m^th^ particle and the swarm, respectively, *w* is the inertia coefficient, *c*_1_ and *c*_2_ are positive constants and *e*_1_ and *e*_2_ are random coefficients between 0 and 1 to guarantee the random behavior of the optimization algorithm^29^. While in (2), *v*_*m*,*n*_ gets real values, in the BPSO algorithm, the position vectors are binary-valued (i.e., *x*_*m*,*n*_ is 1 or 0). Therefore, to map real-valued velocities to binary-valued positions, the sigmoid limiting transformation,

3 $$S({v}_{m,n}^{t})=\frac{1}{1+{{\rm{e}}}^{-{v}_{m,n}^{t}}},$$

is commonly used. Then, the position of the m^th^ particle at t + 1^th^ iteration is updated as

4 $$\begin{array}{rcl}{x}_{m,n}^{t+1} & = & \{\begin{array}{cc}\mathrm{1,} & {r}_{m,n}^{t} < S({v}_{m,n}^{t})\\ \mathrm{0,} & {r}_{m,n}^{t}\ge S({v}_{m,n}^{t})\end{array},\end{array}$$

where ${r}_{m,n}^{t}$ is a random number between 0 and 1.

To evaluate each candidate solution, a fitness function is defined as

5 $$FF=F{F}_{1}+F{F}_{2}$$

where *FF*_1_ and *FF*_2_ are the contributions of harvesting performance at the first and second frequency bands of interest, respectively, defined by:

6 $$\begin{array}{ccc}F{F}_{1} & = & \{\begin{array}{cc}{\eta }_{1}, & {\eta }_{1} < 0.9\\ 0.9, & {\eta }_{1}\ge 0.9\end{array},\end{array}$$

and

7 $$\begin{array}{ccc}F{F}_{2} & = & \{\begin{array}{cc}{\eta }_{2}, & {\eta }_{2} < 0.9\\ 0.9, & {\eta }_{2}\ge 0.9\end{array},\end{array}$$

and *η*_1_ and *η*_2_ are the harvesting efficiencies (defined in (1)) at 2.45 GHz and 6 GHz, respectively. Notice that, in (6 and 7), the fitnesses are defined as a two-criterion function because the design goal was to achieve a harvesting efficiency greater than 0.9 (corresponding to 90%) in both frequency bands of interest. In other words, according to this definition of the fitness function, a design providing a harvesting efficiency of 0.9 and 0.85 at 2.45 GHz and 6 GHz, respectively, is superior than another design with a harvesting efficiency of 1 and 0.8.

As indicated in Fig. 4, to obtain the harvesting efficiency, the MATLAB optimization code is linked to the CST full-wave EM simulator. First, a pre-simulation module which is implemented in MATLAB decodes the string of the bits to a simulation model (i.e. the pattern of the cell) which is imported to the CST software. Then, full-wave simulation is performed using CST by truncating the unit cell by a periodic boundary condition and exciting the cell by Floquet ports in a way that a plane wave normally illuminates the structure. Finally the harvesting efficiencies at the frequencies of 2.45 GHz and 6 GHz are exported to the MATLAB optimization code for evaluation of the fitness function.

By setting the swarm size to 70, the BPSO algorithm converged after approximately 100 iterations. Using the numbering indicated in Fig. 3, the optimal values of the bits were obtained as,

8 $$\beta =\,\begin{array}{c}[0\,0\,0\,0\,0\,1\,1\,1\,0\,0\,0\,1\,1\,1\,0\,0\,0\,0\,1\,1\,1\,1\,0\,0\,1\,1\,1\,0\,0\,0\,1\,1\,1\,1\,1\,1\,1\,0\,0\,0\,0\,0\,1\,1\,0\,1\,1\,0\,1\,1\,0\,0\,0\\ 0\,1\,0\,0\,0\,1\,0\,0\,1\,0\,0\,1\,0\,1\,1\,1\,0\,0\,1\,1\,0\,0\,0\,1\,1\,0\,1\,1\,1\,0\,0\,0\,1\,1\,0\,0\,1\,0\,1\,1\,0\,1\,1\,0\,1\,0\,0\,0\,1\,1\,1\,1]\end{array}$$

**Figure 5:**
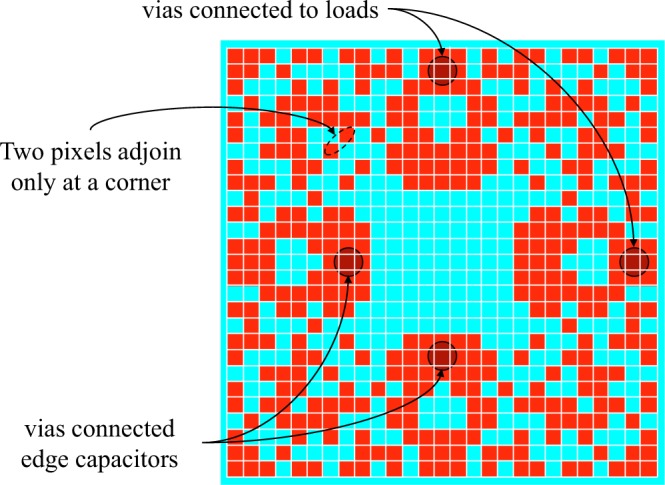
The pixelated pattern resulted by the BPSO algorithm.

**Figure 6:**
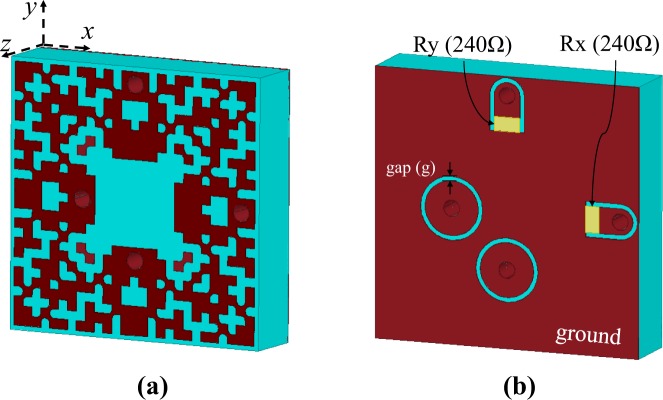
The pixelated unit cell after fabrication considerations, (**a**) top view and (**b**) bottom view ((**b**) is a mirror image of the actual view).

Figure 5 shows the pattern of the cell (the top layer pattern) resulted by the BPSO algorithm. The optimized values of load resistance and gap size were obtained as *R* = 240 Ω and *g* = 0.25 mm, respectively. In the pattern shown in Fig. 5, there are some pixels which adjoin to each other only at a corner. However, in fabrication (photolithography) process, these pixels can be disconnected due to corrosion by etching acid which may result in failure of the design. To solve this fabrication problem, the corner junctions are widened as shown in Figure 6. 3D views of the optimized pixelated unit cell after fabrication considerations are provided in Fig. 6. Notice that the dielectric substrate is the same as that in the preliminary design, i.e. Rogers RT6006 with a thickness of 2.5 mm.

**Figure 7:**
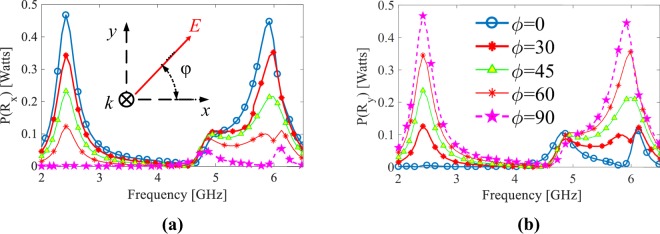
Power delivered to (**a**) Rx and (**b**) Ry shown in Fig. 6(b) for different polarization angles.

By setting the incident power to the cell to 0.5 Watts, Fig. 7(a,b) show the power delivered to Rx and Ry (see Fig. 6(b)), respectively for different polarization angles. When the incident wave is x-polarized (i.e. *ϕ* = 0°), at the operating frequencies of 2.45 GHz and 6 GHz, 0.45 Watts power is delivered to Rx; however, almost no power is delivered to Ry. On the other hand, for the y-polarized incident wave (i.e. *ϕ* = 90°), at the operating, 0.45 Watts power is delivered to Ry while Rx receives almost no power. For the slant polarization angels (i.e. *ϕ* = 30°, 45° and 60°), the captured power by the cell is divides between Rx and Ry in a way that the sum of the delivered power to Rx and Ry at the operating frequencies is still around 0.45 Watts. Importantly, it should be noted that since the cell shown in Fig. 6 has full symmetry, under polarization angle *ϕ* (where 0° ≤ *ϕ* ≤ 90°), the power which is delivered to Rx (Ry) is equal to the power delivered to Ry (Rx) when the polarization angle is 90° − *ϕ*. This is apparent when comparing Fig. 7(a) with Fig. 7(b).

**Figure 8:**
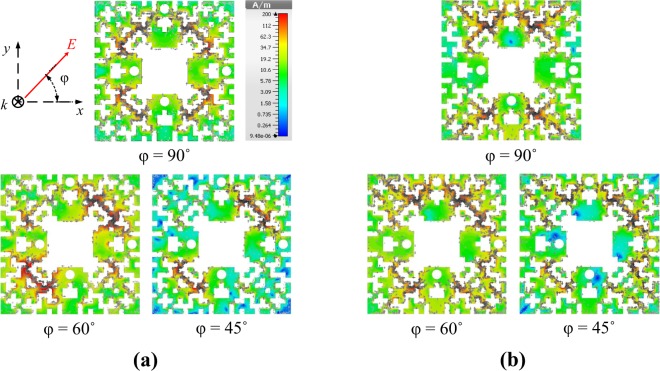
Surface current distribution on the pixelated unit cell at 2.45 GHz (**a**) and 6 GHz (**b**) for different polarization of incident wave.

Figure 8(a,b) depict the surface current distribution on the pixelated resonator (unit cell) at the operating frequencies of 2.45 GHz and 6 GHz, respectively, for different polarizations of incident wave, i.e., *ϕ* = 90°, 60° and 45°. Notice that due to the symmetry of the cell, the current distribution for *ϕ* = 0° and 30° is symmetrical (with 90° rotation) to that of *ϕ* = 90° and 60°, respectively. The figures clearly show that when the electric field is along the y axis (i.e., *ϕ* = 90°), anti-circulating currents on the right and left sides of the resonator merge at the top of the cell and are directed to the load through the top via. For the case of the slant polarized incident wave, i.e., *ϕ* = 45°, the current emanates from the right hole (right load) and sinks into the top via, thus the absorbed power divides between the two loads. Notice that the left and bottom vias are not connected to the loads.

**Figure 9:**
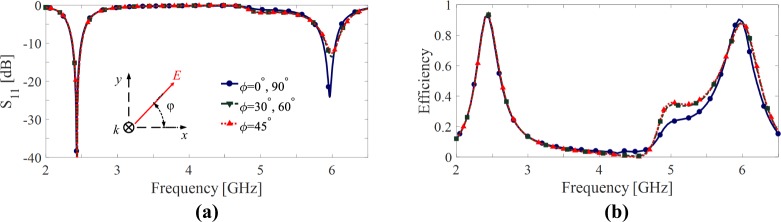
Reflection coefficient (**a**) and harvesting efficiency (**b**) of the pixelated unit cell shown in Fig. 6 for different polarization angles.

Figure 9(a) shows the magnitude of the reflection coefficient (*S*_11_) at the interface between a slab composed of an infinite periodic array of the pixelated unit cell shown in Fig. 6 and the free space for different polarization angles (*ϕ*) of the incident wave. Clearly, for every polarization angle at the frequencies of 2.45 GHz and 6 GHz, the slab is well-matched to the free space such that |*S*_11_| < −13 dB which results in an absorption higher than 95%. The harvesting efficiency of the cell is shown in Fig. 9(b). According to this figure, (1) the pixelated unit cell provides a harvesting efficiency of almost 95% and 90% at the frequencies of 2.45 GHz and 6 GHz, respectively and (2) the harvesting performance of the cell is fully independent from the polarization of incident wave. Therefore, the design objectives have been achieved.

**Figure 10:**
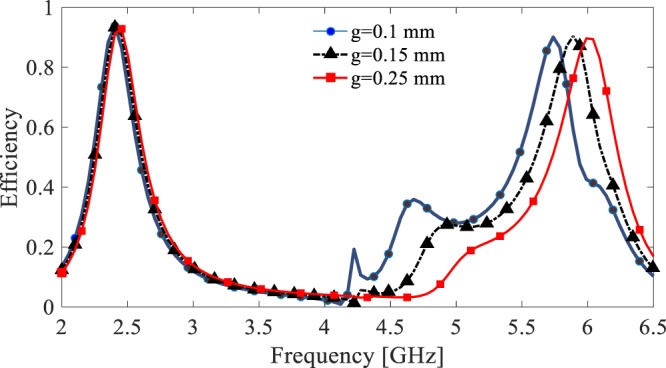
The harvesting efficiency of the pixelated unit cell for different gap size, *g* (see Fig. 6).

It should be mentioned that, as shown in Fig. 10, the simulation result demonstrate that the second operating band of the pixelated unit cell can be well tunned by varying the gap size (*g*). According to this graph, by changing the gap size, one can easily tune the second frequency band with neither affecting the first frequency band nor degrading the harvesting performance.

**Figure 11:**
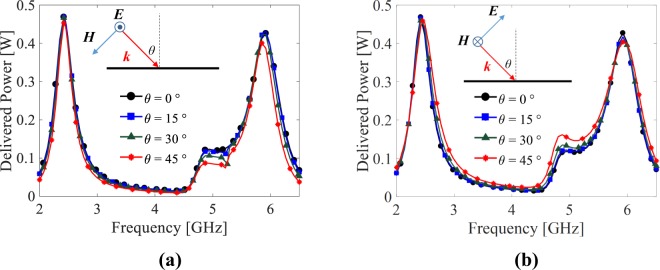
Power delivered to the loads of the unit cell under oblique incidence. (**a**) TE mode and (**b**) TM mode.

Finally, the harvesting performance of the designed pixelated cell is investigated under oblique incidence. To this end, by applying a periodic boundary condition, the unit cell was illuminated by an obliquely incident plane wave where the incidence angle was swept from 0° to 45° in steps of 15°. By setting the incident power to 0.5 Watts, the simulations were performed for both TE (where the electric field is perpendicular to the plane of incidence) and TM (where the magnetic field is perpendicular to the plane of incidence) modes. Figure 11 shows the total power delivered to the two loads of the cell. It is obvious that the optimized cell is very insensitive to the angle of incidence in a way that the delivered power to the loads are almost identical.

## Experimental Verification

Once the unit cell is designed, we consider a 9 × 9 array of the pixelated cell. The simulation results were obtained using the CST software wherein an array of 9 × 9 cells was illuminated by a plane wave having an electric field strength of |**E**_*inc*_| = 1 V/m (equivalent to a power density of *S*_*inc*_ = 0.5/(*η*_0_)) and incident normally on the harvester surface.

**Figure 12:**
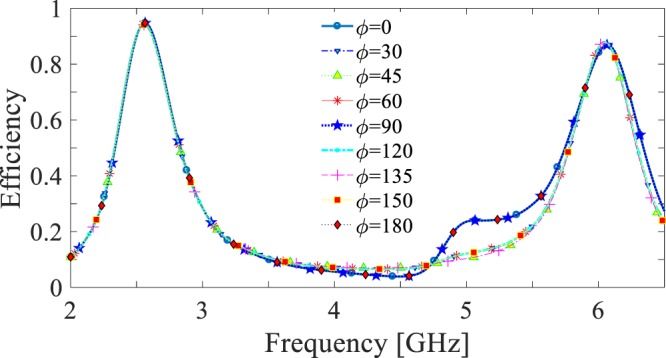
Harvesting efficiency of 9 × 9 array for different polarization angles.

The harvesting efficiency of the finite array can be calculated as the ratio of the total power delivered to the 162 loads (*P*_*loads*_) to the incident power to the array (*P*_*inc*_). Considering a normally incident plane wave, *P*_*inc*_ is calculated as

9 $${P}_{inc}=\frac{1}{2{\eta }_{0}}{|{{\bf{E}}}_{inc}|}^{2}{A}_{array},$$

where *η*_0_ is the characteristic impedance of free space and *A*_*array*_ = 81 *A*_*cell*_ is the physical area of the array (equal to 81 times of the physical area of a cell)^19–21,35,36^. Figure 12 shows the simulated total harvesting efficiency of the 9 × 9 array of the pixelated cell for different polarizations of incident wave. In comparison with Fig. 9(b), it is obvious that the total harvesting efficiency of the finite array as well as the efficiency of the unit cell (i.e., an infinite array) satisfy the goals of the design of having a harvesting efficiency of equal or greater than 90% at both frequencies of interest irrespective of the polarization of the incident wave.

**Figure 13:**
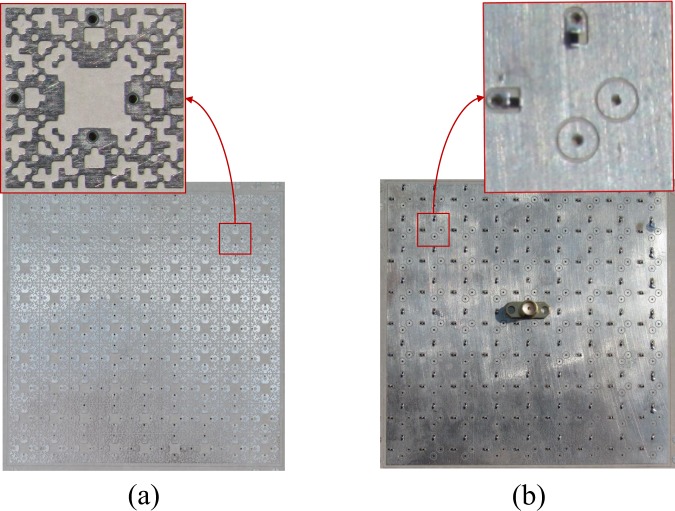
Fabricated array of 9 × 9 optimized unit cell.

**Figure 14:**
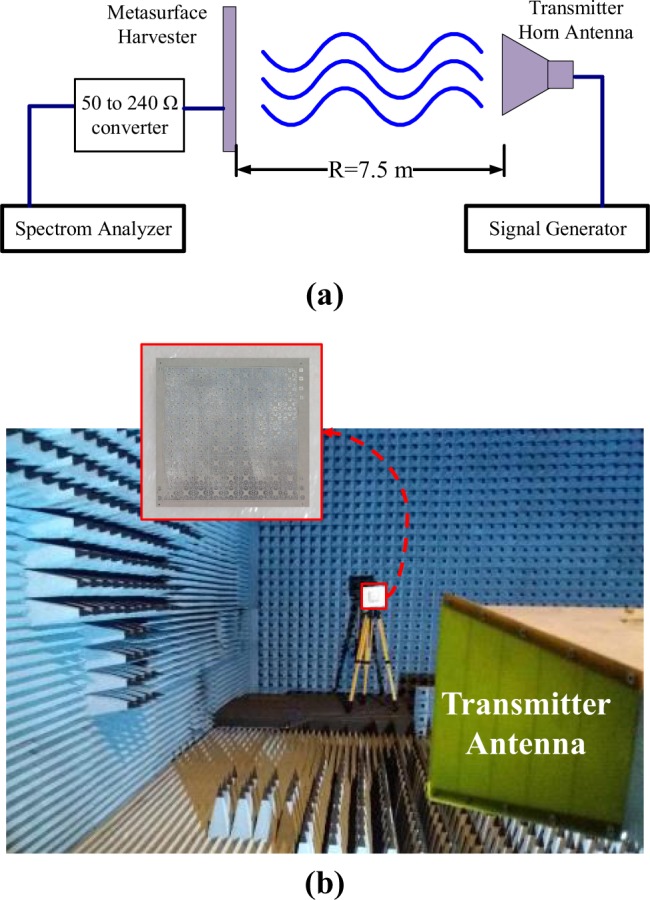
(**a**) A schematic showing the measurements setup and (**b**) the actual measurement setup inside an anechoic chamber.

As shown in Fig. 13, the 9 × 9 array of the pixelated cells was fabricated where each cell was loaded with two identical 240 Ω resistors. By setting up a measurement system in an anechoic chamber (see Fig. 14), the output power delivered to the loads of the central cell was measured for different polarization angles of the incident field. A resistor of the central cell was replaced by an SMA connector (see Fig. 13(b)). Since the input impedance of most instruments is 50 Ω, to overcome the impedance mismatch between a 50 Ω instrument and the harvester, a 50 Ω to 240 Ω impedance converter was placed between the SMA connector and a 50 Ω spectrum analyzer used for measurements (see Fig. 14(a)). To prevent the 50 Ω connection between the cell and the impedance converter introducing an impedance mismatch, by proper cabling, the length of the connection was precisely set to half a wavelength at each operating frequency, i.e. 2.45 GHz and 6 GHz (two different connection lengths). This issue restricted our measurement to the vicinity of the operating frequencies. The distance between the transmitter horn antenna (R&S HF906^37^) and the harvester was 7.5 m to ensure that the board was excited by a plane wave. A cross line laser was used for precise leveling (positioning) of the harvester.

**Figure 15:**
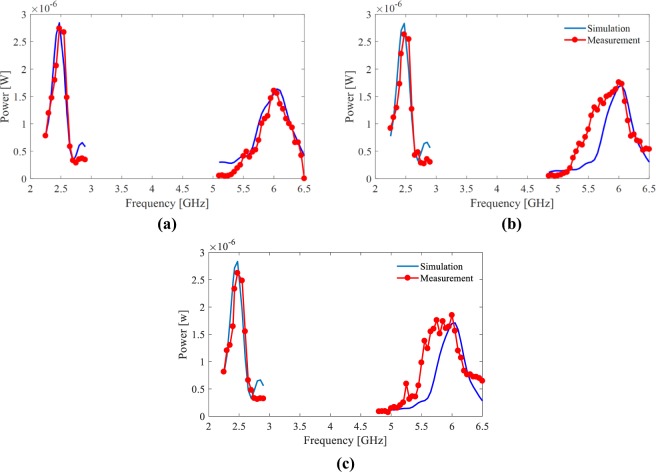
Simulated and measured power delivered to the terminals of the central cell for different polarization angles of incident wave, (**a**) 0° and 90°, (**b**) 30° and 60° and (**c**) 45°.

Figure 15 shows the simulated and measured output power (the power delivered to the two loads) of the central cell for different polarization angles of the incident field, 0° (and 90°), 30° (and 60°) and 45°. For each polarization angle, the simulation result was obtained by adding the power delivered to Rx and Ry of the central load. However, Since the power which is delivered to Rx (Ry) under polarization angle *ϕ* is equal to the power delivered to Ry (Rx) when polarization angle is 90° − *ϕ*, the measured power delivered to the two loads of the central cell for a polarization angle of *ϕ* was evaluated as the sum of the power delivered to one port (Ry) when the polarization angle is *ϕ* and 90° − *ϕ*. To have a fair comparison between the simulation and measurement results, in the measurements, the transmitted power was set such that the power density of the incident wave at the array under test was the same as that in the simulations. This was achieved using the Friis transmission equation,

10 $${S}_{inc}=\frac{{P}_{t}\,{G}_{t}}{4\pi {R}^{2}},$$

where *P*_*t*_ is transmitted power, *G*_*t*_ is transmitter antenna gain and *R* is distance between the transmitter antenna and the harvester array. A strong agreement between the simulated and measured results is observed in Fig. 15 which validates our simulations.

Notice that, Fig. 15 suggests that in comparison with the frequency of 6 GHz, considerably more power is delivered to the loads of the central cell at the frequency of 2.45 GHz. However, according to Fig. 12, almost the same total power at both frequencies is expected. Specifically, our simulation results show that, although in the central cells of a finite array the delivered power (to the two loads) in the first frequency band is higher, the outer cells compensate the shortage of delivered power in the second frequency band in a way that, as depicted in Fig. 11, the entire array provides an almost same amount of delivered power at both frequencies. For a very large array, say 100 × 100; however, we would expect that most cells would absorb higher power at the lower frequency as the number of cells close to the periphery of the array will be a small percentage of the total number of cells.

## Conclusion

In a conclusion, we presented the design of a novel metasurface for dual-band and multi-polarization electromagnetic energy harvesting. The proposed metasurface is composed of unit cells which was designed by applying a binary optimization algorithm to the pixelated area of a cell and linking it to a full-wave EM simulator. Using this highly robust and fully automated unit cell design approach, a harvesting efficiency of greater than 90% at both frequencies of interest (which was set to 2.45 GHz and 6 GHz) was achieved. The simulation results showed that the optimized unit cell was not only insensitive to the polarization of a normally incident wave but also to the polarization and incident angle of an obliquely incident plane wave. As a proof of concept, an array of 9 × 9 cells was simulated, fabricated and tested in an anechoic chamber. The measured and simulated results were in a strong agreement. It was shown that the array provides a harvesting efficiency of close to or greater than 90% in both the frequency bands irrespective of the polarization of the incident wave. Finally, we note that due to the low impedance values of the terminals in comparison to the designs in earlier works, the metasurface designed here is more suitable for application in real-world harvesting systems.
